# *QuickStats:* Percentage of Persons in Families Having Problems Paying Medical Bills in the Past 12 Months,* by Age Group — National Health Interview Survey, 2011–2017^†^

**DOI:** 10.15585/mmwr.mm6814a6

**Published:** 2019-04-12

**Figure:**
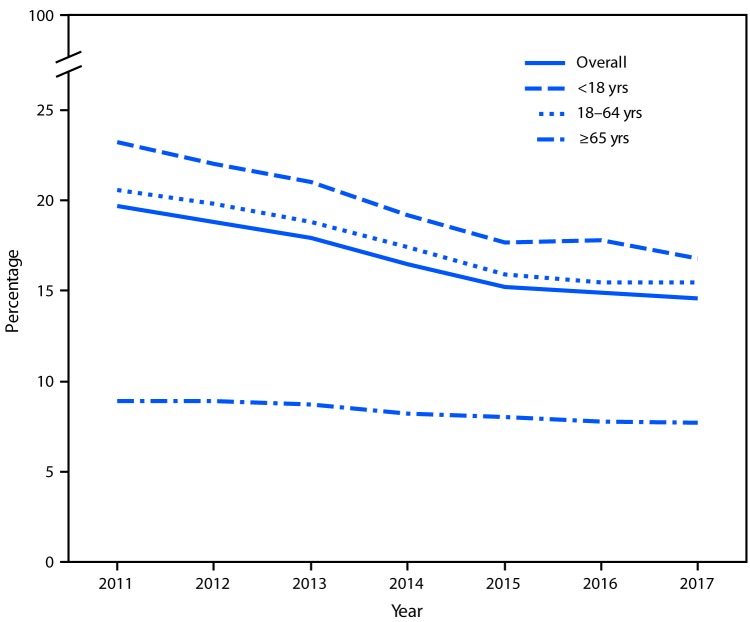
From 2011 to 2017, the overall percentage of persons who were in U.S. families having problems paying medical bills in the past 12 months decreased from 19.7% to 14.6%. Similar trends were observed for all age groups, with a decrease from 23.2% to 16.8% for children aged <18 years, from 20.6% to 15.5% for adults aged 18–64 years, and from 8.9% to 7.7% for those aged ≥65 years.

**Source:** National Health Interview Survey, 2011–2017 data. https://www.cdc.gov/nchs/nhis.htm.

